# Rapid loss of glacial ice reveals stream community assembly processes

**DOI:** 10.1111/j.1365-2486.2012.02675.x

**Published:** 2012-03-26

**Authors:** Lee E Brown, Alexander M Milner

**Affiliations:** * School of Geography/water@leeds, University of LeedsLeeds, LS2 9JT, UK; † School of Geography, Earth and Environmental Sciences, University of BirminghamBirmingham, B15 2TT, UK; ‡ Institute of Arctic Biology, University of AlaskaFairbanks, AK, 99775, USA

**Keywords:** assembly, deterministic, Glacier Bay, macroinvertebrate, niche filtering, river, stochastic, traits

## Abstract

Glacial retreat creates new habitat which is colonized and developed by plants and animals during the process of primary succession. While there has been much debate about the relative role of deterministic and stochastic processes during terrestrial succession, evidence from freshwater ecosystems remains minimal and a general consensus is lacking. Using a unique 27 years record of community assembly following glacial recession in southeast Alaska, we demonstrate significant change in the trait composition of stream invertebrate communities as catchment glacial cover decreased from ∼70% to zero. Functional diversity increased significantly as glacier cover decreased and taxonomic richness increased. Null modelling approaches led to a key finding that niche filtering processes were dominant when glacial cover was extensive, reflecting water temperature and dispersal constraints. Thereafter the community shifted towards co-occurrence of stochastic and deterministic assembly processes. A further novel discovery was that intrinsic functional redundancy developed throughout the study, particularly because new colonizers possessed similar traits to taxa already present. Rapid glacial retreat is occurring in Arctic and alpine environments worldwide and the assembly processes observed in this study provide new fundamental insights into how glacially influenced stream ecosystems will respond. The findings support tolerance as a key primary successional mechanism in this system, and have broader value for developing our understanding of how biological communities in river ecosystems assemble or restructure in response to environmental change.

## Introduction

Climate change is causing glacial retreat to proceed rapidly across the world (Dyurgerov & Meier, 2006; Milner *et al*., 2007), leading to the creation of new habitats which will be colonized and developed by plants and animals during the process of primary succession (Chapin *et al*., 1999; Fastie, 2000; Kaufmann, 2001). Successional changes over decades to centuries are known to be driven by both biotic (e.g. facilitation, inhibition, tolerance) and/or abiotic (e.g. temperature, moisture, pH) processes (Fastie, 2000; Engstrom *et al*., 2006), and the clear changes in biological assemblages during succession have underpinned ongoing major debates around the general principles, or assembly rules, which underpin community development (e.g. MacArthur & Levins, 2007; Connor & Simberloff, 2007; Lawton, 2001; Weiher & Keddy, 2006; Hubbell, 2006; Purves & Turnbull, 1997).

Attempts to reveal the assembly processes that structure biological communities have largely used approaches based on trait diversity (Petchey & Gaston, 2005). Functional diversity indices take into account redundancy among species that assume similar functional roles (Petchey *et al*., 2002), and so observed functional diversity for a biological community can be compared against null (stochastic or ‘neutral’ – Hubbell, 2006) models constructed from the regional species pool (Weiher & Keddy, 2006; Petchey *et al*., 2002) to investigate assembly processes. Significant departures from stochastic models may indicate either deterministic competitive exclusion/limiting similarity (observed > random), or environmental/niche filtering (observed < random), to be important determinants of local assemblage structure (Cornwell *et al*., 1979; Holdaway & Sparrow, 2007). In reality though, these assembly mechanisms have been shown to occur simultaneously, or even switch, along environmental gradients (Mason *et al*., 1987; Thompson & Townsend, 2010; Helmus *et al*., 1983). While null modelling of functional diversity has been applied to various biotic groups and habitats to test hypotheses of community assembly for ‘mature’ communities (Stubbs & Wilson, 2007; Micheli & Halpern, 1967; Petchey *et al*., 2002), the approach has so far not been applied to ecosystems undergoing the early stages of primary succession, for example where glaciers have retreated and exposed new terrain. In these situations, assembly processes can be monitored directly over time as the environment changes year on year to provide insights that may be missed in space-for-time substitution studies (Johnson & Miyanishi, 2006).

Glacial retreat in Alaska has been occurring since around 1750 (end of the Little Ice Age), opening up extensive areas of deglaciated terrain, and hundreds of new streams undergoing primary succession have been created (Milner, 2005). Since the late 1970s, particularly extensive and rapid reductions in glacial mass have been observed for many of the low elevation glaciers along the Gulf of Alaska (Larsen *et al*., 2008). In 1978, a detailed study of one such stream, Wolf Point Creek (WPC) in Glacier Bay, southeast Alaska, was initiated. At that time, WPC was sourced from a basin with ∼70% glacial ice cover. Sampling has since been undertaken in late August/early September during the intervening years up to 2004 when the remnant glacial ice had disappeared completely. Progressive increases in stream temperature, riparian vegetation cover and habitat complexity over time have been accompanied by colonization of a diverse group of invertebrates (Milner *et al*., 2001). In 1978, when catchment glacial cover was ∼70%, only Chironomidae (non-biting midge larvae) of the sub-families Diamesinae and Orthocladiinae, able to complete their life cycle in water temperature <2 °C, were found. Ephemeroptera (mayfly) and Plecoptera (stonefly) larvae appeared at ∼50–60% glacierization in 1986 and the first non-insect taxa (Oligochaete worms) at ∼30% glacierization in 1992. Dytiscidae (diving-beetles) and Corixidae (water-boatmen) were first collected in 2000 and 2003, respectively, after ice masses had almost vanished (Milner *et al*., 2007). However, all of our previous investigations in the Wolf Point Creek system have been based on taxonomic analyses, and so the processes of community assembly have not been investigated in detail.

This paper provides new insights into community assembly using the WPC long-term primary succession record by: (i) providing the first study of changes in stream macroinvertebrate traits as stream ecosystems evolve following glacial retreat; (ii) investigating how functional diversity responds to glacier retreat and changes in taxonomic diversity, and; (iii) applying null modelling approaches to determine how deterministic and/or stochastic processes might influence community assembly in freshwater ecosystems. The use of multiple traits to characterize the functional composition and diversity of stream invertebrate communities (cf. focus on taxonomic structure) is well established in the ecological literature (Statzner & Bêche, 2002) but, while space-for-time substitutions have been used to infer stream macroinvertebrate trait responses to glacial influence (Snook & Milner, 2001; Ilg & Castella, 2003; Füreder, 2010), no published studies have considered how trait composition and functional diversity change over time as glaciers retreat and disappear.

We tested three hypotheses to gain an improved understanding of the patterns and processes of community assembly during primary succession: (H_1_) functional diversity of the stream macroinvertebrate community will be lower than randomly generated communities during the early stages of primary succession, because environmental filters (e.g. low water temperature, unstable substratum, dispersal constraints) select for traits (e.g. cold stenothermic thermal preference, short life-cycles, long-range dispersal ability) that allow taxa to colonize, survive and reproduce effectively in this environment; (H_2_) as primary succession proceeds, functional diversity of the stream community reflects random assembly processes, because environmental filtering decreases in importance as water temperature and habitat heterogeneity increase, and stochastic colonization and disturbance events (e.g. rainfall-induced bed disturbances) become more important and; (H_3_) functional redundancy is prevalent within the stream macroinvertebrate community, particularly during the early stages of primary succession, because environmental filtering yields taxa with similar traits (Cornwell *et al*., 1979). The findings from the study are considered in relation to general theories of primary succession, ecosystem responses to glacier retreat and other long-term studies of biological communities.

## Methods

### Site description and sampling

The mouth of WPC (58°59′49.84″N, 136°9′57.05″W) was uncovered from glacial ice in the mid-1940s and the stream is now approximately 2 km in length, 6–10 m wide, and flows over glacial moraine, till, and outwash deposits. Lawrence Lake (unofficial name), which feeds the stream, emerged in the early 1970s and is currently 1.45 km^2^ in size, with a maximum depth of 35 m. In 1978, the lower floodplain was essentially barren and aerial photographs indicate that the catchment was ∼70% glacierized. Isolated clumps of alder (*Alnus crispa*) and willow (*Salix* spp.) were evident on upper terraces, where mats of mountain aven (*Dryas* spp.) were nearly continuous. By 1988, the lower terraces supported a few clumps of alder and willow and glacial cover had decreased to ∼50%. Dolly Varden (*Salvelinus malma*) were the first fish to colonize in 1987, followed by pink (*Oncorhynchus gorbuscha*) and coho (*Oncorhynchus kisutch*) salmon in 1989. By 1997 (<10% glacierization), alder and willow were dominant with riparian plants exceeding 3 m in height and pink salmon numbering >12 000 individuals (Milner *et al*., 2001). In 2004, glacial ice had almost disappeared completely and the upper terraces supported increasing numbers of cottonwood (*Populus trichocarpa*) with the occasional Sitka spruce (*Picea sitchensis*).

From 1978, macroinvertebrates were collected in August or early September (with the exception of 1979–1985, 1987, 1995 and 2003) using a Surber net (ten replicates; 330 μm mesh), from a representative sampling station 0.75 km from the stream mouth. Qualitative hand-search samples were collected from 1979 to 1985 at the same location. Invertebrates were preserved in 70% ethanol and later sorted from detritus and inorganic matter in the laboratory and identified using Merritt *et al*. (2003). Chironomidae larvae were identified using methods outlined in Milner *et al*. (1994). Stream temperature was measured using handheld thermometers and Gemini TinyTag dataloggers (see Milner *et al*., 2001 for details), and turbidity (NTU) measured spectrophotometrically on stream water samples based on APHA standard methods (Clesceri *et al*., 1998). These variables, together with increased riparian vegetation cover and habitat complexity, are considered the central drivers of invertebrate primary succession following deglacierization (Milner *et al*., 2001).

### Data analysis

Pearson's correlation coefficients were calculated to show the association between glacial cover and water temperature and turbidity. Macroinvertebrate community structure was summarized by calculating taxonomic richness and Log_10_ (total abundance + 1). Traits were characterized for 37 taxa using the database of Poff *et al*. (2007) for insects, and for non-insects using the meta-database of traits for North American invertebrates developed by Vieira *et al*. (2003). One taxon from the WPC dataset (Hydracarina) was not included due to a lack of relevant trait information. Taxa were classified according to 20 traits coded in 63 modalities which can be broadly categorized as life-history, mobility, morphological and ecological traits (see supplementary Tables S1, S2). The selected categories encompassed a range of biological and functional states that were expected to respond strongly to ecosystem changes associated with decreasing glacial influence (Snook & Milner, 2001; Ilg & Castella, 2003). In line with previous studies of North American stream macroinvertebrates (Finn & Poff, 2000; Poff *et al*., 2007), each taxon was assigned to one modality per trait (i.e. binary approach). Traits were assigned at genus level for most taxa. Some taxa were identified at family level and for these we used the majority rule approach of Poff *et al*. (2007) to assign the most common genus-level modality. Chironomidae comprised 21/37 taxa (Table S1) and traits were assigned for genera or species wherever possible based on additional published information (e.g. Snook & Milner, 2001; Ilg & Castella, 2003).

Trait data collected each year were ordinated using Non Metric Multidimensional Scaling (NMDS) in PRIMER v6 (Clarke & Chorley, 1994). Arcsin transformed trait relative abundance data, Bray-Curtis dissimilarities and 2000 restarts were used in the analysis. A one-way (between years) similarity of percentages (SIMPER) routine (Clarke & Chorley, 1994) was used to determine which traits accounted for the greatest dissimilarity across the sampling period. For brevity we examined the relative abundance of the top five traits as a function of catchment glacierization using linear regression.

For each year of sampling, the number of traits (richness) represented in the invertebrate community was counted. Functional diversity was estimated first by calculating Functional Diversity (FD: Petchey & Gaston, 2005), a measure of functional richness (Schleuter *et al*., 1982), whereby the taxa by trait matrix was converted into a distance matrix (by calculating Euclidean distance) which was subsequently clustered (UPGMA routine) to produce a functional dendrogram that depicts the functional relationships among the entire macroinvertebrate assemblage of the WPC catchment. FD of the entire WPC assemblage was taken as the total branch length of this dendrogram, and FD of assemblages for different years was the total length of the branches required to connect all of the species in that assemblage, standardized by FD for the entire assemblage. Thus, variation in FD ranged from 0 to 1, where a value of zero represented single species communities or those in which all taxa had identical trait profiles (Petchey & Gaston, 2010).

Second, functional diversity was calculated using Rao's quadratic entropy (QE) (Rao, 2006), a measure of functional divergence (Schleuter *et al*., 1982), where distances between species pairs were first calculated using Bray-Curtis dissimilarities and then integrated with the relative abundance of taxa, to estimate the probability of two randomly selected taxon pairs having the same trait profile.

Studies have suggested that dendrogram methods (e.g. FD: Petchey & Gaston, 2005, 2010) are suitable for studies examining community assembly where taxonomic richness is >10, and the n taxa < n traits used to calculate FD (Mouchet *et al*., 2009). Therefore, FD was used to simulate five scenarios of macroinvertebrate arrival in WPC: (i) random colonization: the taxonomic richness of the observed assemblage for each sampling year was determined, and then that number of taxa was drawn randomly 25 times from the entire taxonomic pool, and FD calculated for each; (ii) colonization in the order that maximized FD (limiting similarity): the taxonomic richness of the assemblage for each sampling year was calculated, and then the combination of taxa providing the maximum possible FD for that level of richness was determined; (iii) colonization in the order that minimized FD (environmental filtering): opposite of step ii; (iv) colonization determined by a taxon's thermal preference: steps i–iii were repeated but from the pool of taxa possessing cold stenothermic traits (10 random draws per level of taxonomic richness), to examine the role of water temperature, a major environmental ‘filter’ influencing macroinvertebrate communities in glacial streams (e.g. Milner *et al*., 1997; Brown *et al*., 2009), and; (v) colonization determined by a taxon's aerial dispersal ability: steps i-iii repeated but from the pool of insects with high (>5 km) adult dispersal distances (10 random draws per level of taxonomic richness), to examine the extent to which delays in colonization by poor dispersers (e.g. short adult flying distance or non-insect taxa lacking aerial stages) due to barriers such as coastal inlets and high mountain ranges (Milner *et al*., 2001), play a role in community assembly. The hand-search samples collected between 1979 and 1985 enabled the inclusion of n taxa = 3 and 4 in the modelling analyses because abundance data are not required to calculate FD.

To examine how successional change in FD of WPC macroinvertebrate assemblages was related to compositional change between sampling years, we compared the observed change in FD to the observed change in taxonomic richness using linear regression (Micheli & Halpern, 1967). This approach provides information on intrinsic redundancy, resulting from functional similarity among taxa (Petchey *et al*., 2002). Assemblages containing many similar taxa have high intrinsic redundancy, and random taxonomic changes have little effect on FD. Second, we compared the observed change in FD to the change in FD predicted at random, using mean data from each of the 25 draws outlined in step i. This approach provides information on extrinsic redundancy, which can result when non-random compositional changes are non-random with respect to functional traits (Petchey *et al*., 2002). Where extrinsic redundancy is high, the loss of relatively unique taxa causes a relatively large decrease in FD.

Linear regression was used to assess relationships between catchment glacier cover and the various dependent stream invertebrate taxonomic and functional diversity indices. All data sets were time-series but examination of Durbin-Watson statistics suggested a lack of autocorrelation and therefore ordinary least squares (OLS) regression was preferred over generalized regression, with relationships considered significant for *P* < 0.05. All variables were tested for normality with Anderson–Darling tests and *P* > 0.05 used to infer normality. All statistical analyses were undertaken in Minitab 15.0 (Minitab, Coventry, UK) and considered significant at *P* < 0.05.

## Results

**Fig. 1:**
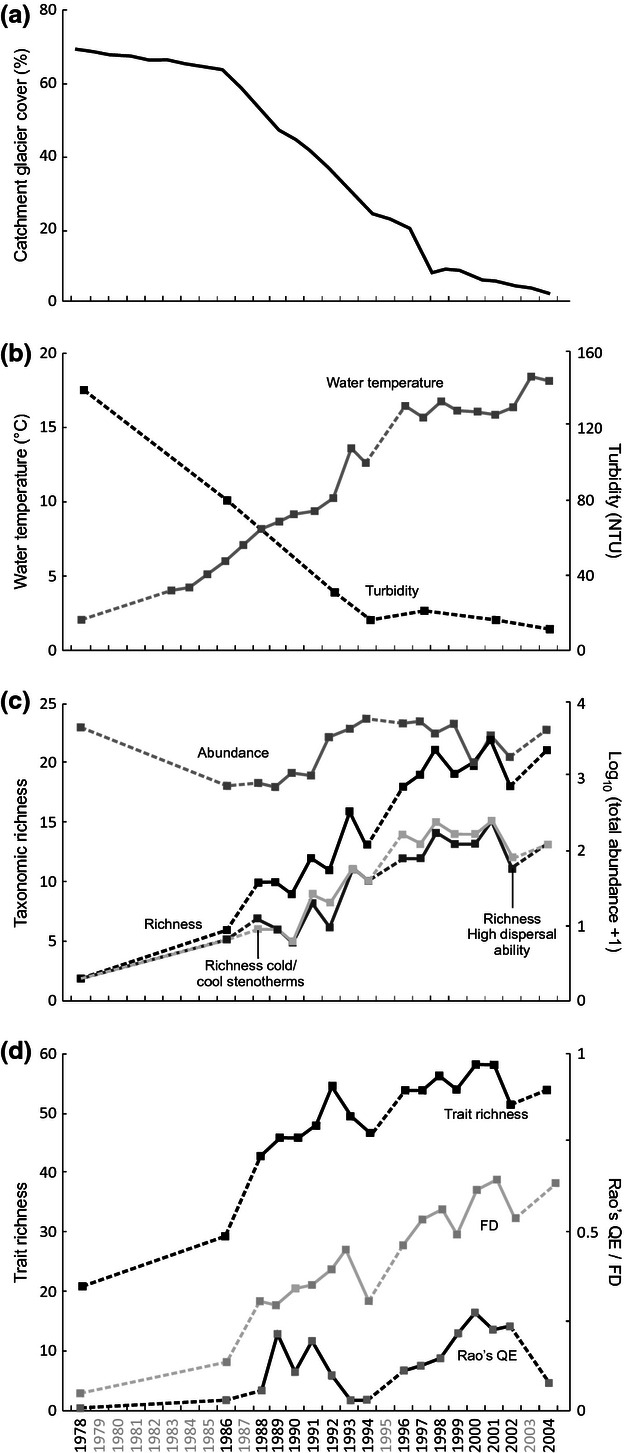
Temporal change at Wolf Point Creek from 1978 to 2004 of: (a) catchment glacierization (%); (b) mean of August daily mean water temperature, and turbidity; (c) log_10_[total abundance + 1], taxonomic richness, richness of cold/cool stenotherms and richness of organisms with high (>5 km) adult dispersal ability; (d) Trait richness, FD and Rao's QE. (Broken lines denote changes between years where quantitative samples for intervening years were not collected).

The consistent decrease in glacial cover over time was accompanied by a significant rise in stream temperature (R^2^ = 0.95, *P* < 0.001) and a decrease in turbidity (R^2^ = 0.95, *P* < 0.001; Fig. 1a and b). Invertebrate richness increased from 1978 to 2004 whereas total abundance was lowest during the late 1980/early 1990s and in 2000 (Fig. 1c). From a possible 63 modalities, the number of traits represented within the invertebrate community (trait richness) at WPC increased from 21 in 1978 to a maximum of 58 in 2000/2001 (Fig. 1d). FD followed a similar pattern to trait richness, increasing over time to peak in 2001 and then decreasing slightly to 2004. Rao's QE also showed a general increase over time but fluctuations were more pronounced, with peaks in the early 1990s and around 2000 interspersed by lower values (Fig. 1d).

**Fig. 2:**
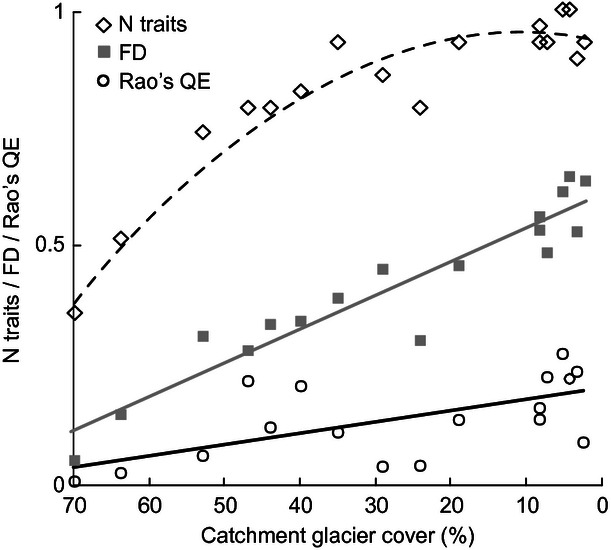
Regression of functional diversity against catchment glacier cover (%). Table 1 provides regression summaries. (N traits was standardized from 0 to 1 by dividing by the maximum number of traits observed).

All measures of taxonomic and functional diversity were associated significantly with decreases in catchment glacierization (Table 1; Fig. 2). The NMDS produced a best-fit configuration with three dimensions (stress = 0.02) which represented 99% of the total variance. The two-dimensional solution had a stress of 0.05. Although many trait modalities were strongly inter-correlated, the SIMPER analysis highlighted that high dispersal ability, adult ability to exit the water, cold or cool stenotherms, sprawling habit and univoltine life cycle accounted for 23.5% of the dissimilarity. All five traits showed relatively weak but significant positive association with catchment glacial cover, with the strongest relationship observed for invertebrates with sprawling habit (Table 1).

**Table 1:** Regression analysis summary results for dependent taxonomic and trait variables against per cent catchment glacier cover. (Response denotes association of dependent variable with decreased glacial cover)

Dependent variables	df	R^2^	*P*	Response
Taxonomic				
Taxonomic richness	16	0.95	<0.0001	↑
Log_10_ total abundance	16	0.24	0.05	↑
Trait				
N traits	16	0.91	<0.0001	↑
Rao's QE	16	0.35	0.01	↑
FD	18	0.81	<0.0001	↑
Adult ability to exit water	16	0.26	0.04	↓
Cold or cool stenotherm	16	0.25	0.04	↓
High dispersal ability	16	0.25	0.04	↓
Sprawling habit	16	0.48	0.002	↓
Univoltine	16	0.28	0.03	↓

**Fig. 3:**
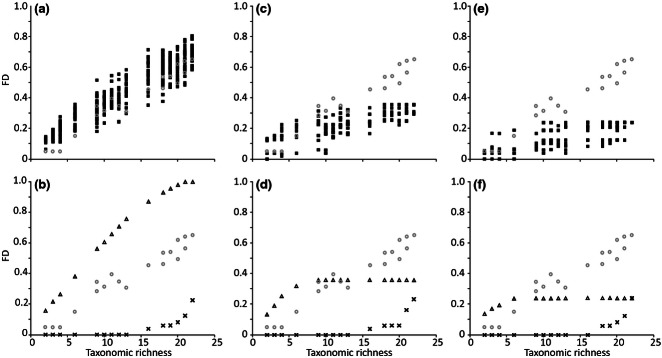
Observed FD (open grey circles), randomly generated FD (black filled squares), FD under the limiting similarity assembly rule (open triangles) and FD under the niche filtering assembly rule (black crosses) as a function of taxonomic richness for (a) and (b) the entire Wolf Point Creek macroinvertebrate assemblage, (c) and (d) assemblages generated only from cold stenothermic taxa, and (e) and (f) assemblages generated only from insect taxa with high (>5 km) adult dispersal ability.

During the initial stages of primary succession, FD remained unchanged when taxonomic richness was between two and four taxa (Fig. 3a), but overall a strong and significant linear relationship between taxonomic richness and FD was evident (Table 2). FD of early successional communities was lower than expected by chance and more similar to the environmental filtering model than the limiting similarity model (Fig. 3b). As primary succession proceeded and taxonomic richness increased, observed FD overlapped strongly with FD for randomly generated communities (Fig. 3a), and became intermediate between niche filtering and limiting similarity assembly models.

**Table 2:** Summary statistics for linear regression models between observed FD, expected FD and observed taxonomic richness. (nb. all relationships were positive; cf. Fig. 4)

Dependent variable	Independent variable	df	Intercept (±1 SE)	Slope	R^2^	*P*	Interpretation
Observed FD	Observed taxonomic richness^1^	18	−0.006 (±0.03)	0.64 (±0.04)	0.94	<0.0001	Slope <1 indicates functional redundancy
Observed FD	Expected FD	18	−0.10 (±0.03)	1.08 (±0.06)	0.95	<0.0001	Observed FD significantly lower than expected FD
Observed change in FD	Observed change in taxonomic richness	169	0.001 (±0.008)	0.62 (±0.02)	0.85	<0.0001	High intrinsic redundancy
Observed change in FD	Expected change in FD	169	0.001 (±0.008)	1.04 (±0.03)	0.86	<0.0001	Absence of extrinsic redundancy

1 Observed taxonomic richness standardized to 0, 1.

The colonization scenario determined by thermal preference included 24 cold-stenotherms from the 36 taxa collected in WPC. Random communities generated from these 24 taxa were characterized by FD highly similar to the real community until richness exceeded 13 taxa (Fig. 3c). For assemblages of cold stenothermic taxa, functional redundancy was evident when taxon richness exceeded nine, as exemplified by the asymptote of the limiting similarity model (Fig. 3d). The colonization scenario determined by an ability to disperse >5 km in the adult aerial stage included 22 of the 36 taxa present in WPC. Random communities generated from these 22 taxa were characterized by FD highly similar to the real community until richness exceeded nine taxa (Fig. 3e). For assemblages of taxa with high dispersal ability, functional redundancy occurred when taxon richness exceeded six, as exemplified by the asymptote of the limiting similarity model (Fig. 3f).

**Fig. 4:**
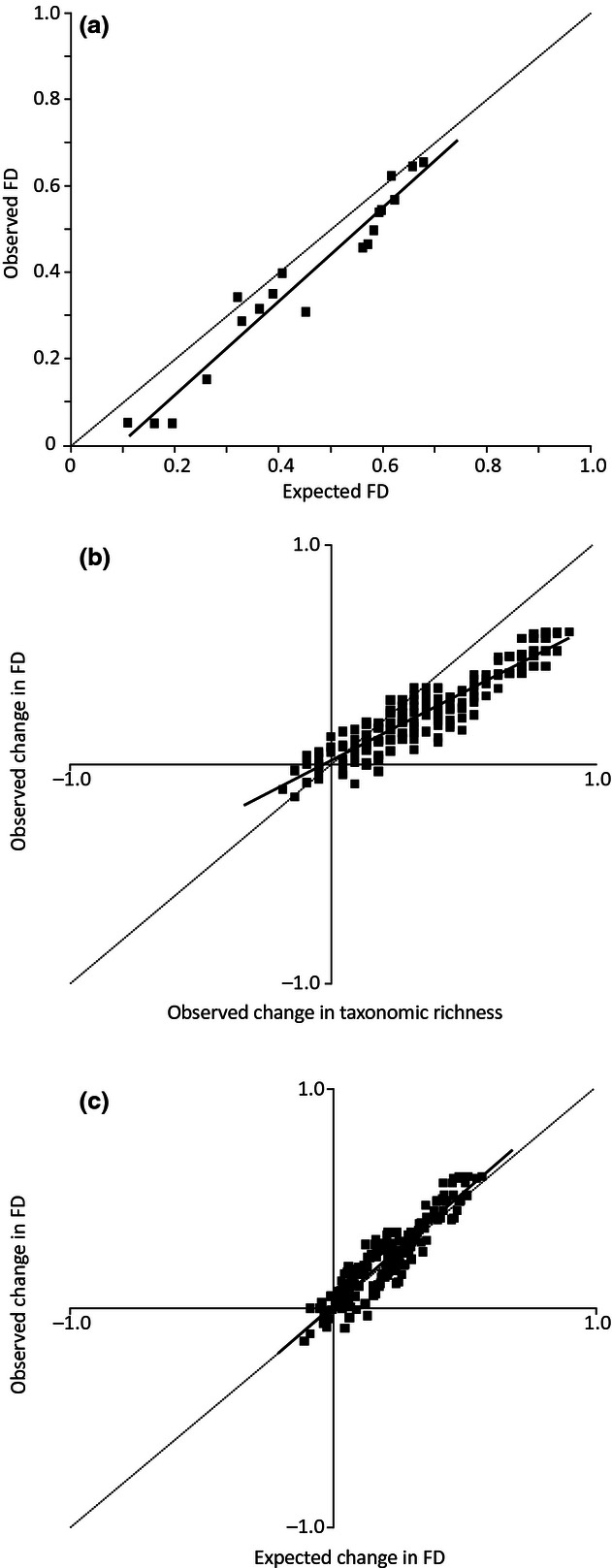
Relationship between (a) expected FD (mean of random assemblages) and observed FD; (b) observed change in taxonomic richness (standardized to 0, 1) and observed change in FD, and; (c) expected and observed changes in FD. Solid black lines denote the linear regression model relationship and broken lines denote the 1 : 1 relationship.

Observed FD for the stream macroinvertebrate community was typically lower than expected by chance as shown by the relationship between observed FD and mean expected FD (Fig. 4a; Table 2). However, as taxonomic richness increased with primary succession the observed FD became more similar to expected FD as shown by the regression slope of 1.08 (±0.06). Strong and significant relationships were evident between observed changes in FD, taxonomic richness and expected changes in FD (Table 2). The increase in FD was proportionally lower than the increase in taxonomic richness, with a slope of 0.62 (±0.02) indicating high intrinsic redundancy (Fig. 4b). Change in FD was marginally higher than would be expected if colonization events were random with respect to functional traits (Fig. 4c) but the slope of 1.04 (±0.03) indicated a lack of extrinsic redundancy.

## Discussion

This study has demonstrated long-term changes in the biological trait composition and functional diversity of aquatic macroinvertebrate communities following glacial recession. While some previous studies in Europe have examined spatial patterns of invertebrate traits in relation to catchment glacier cover (Snook & Milner, 2001; Ilg & Castella, 2003; Füreder, 2010), our study of WPC offers an alternative viewpoint by tracking changes in trait composition and functional diversity over a prolonged time-period as an ice mass has ablated and disappeared. The analysis also provides novel insights into biological trait dynamics of a glacially influenced stream in North America for comparison with previous European studies.

### Stream ecosystem dynamics following glacial retreat

As primary succession has progressed, the WPC watershed has shifted from one dominated solely by Diamesinae (Chironomidae) to a more diverse assemblage which includes Ephemeroptera (mayflies), Plecoptera (stoneflies), Trichoptera (caddisflies) and Coleoptera (beetles) (Milner *et al*., 2001). These taxonomic changes are similar to other studies that examined community response to changing glacial influence on stream ecosystems (Robinson *et al*., 2010; Brown *et al*., 2009; Finn *et al*., 1995). However, only a handful of studies have examined invertebrate biological trait dynamics in glacier-fed rivers (Snook & Milner, 2001; Ilg & Castella, 2003; Füreder, 2010), and these have all been undertaken in Europe using space-for-time substitution methods. The number of biological traits represented in the community and two other measures of functional diversity (FD and Rao's quadratic entropy) showed significant increases over time as glacial cover decreased, and a greater diversity of macroinvertebrate taxa colonized the stream. In contrast to Bêche *et al*. (2006) and Bêche & Resh (2007) who studied non-glacial rivers and found trait diversity to be relatively invariable despite changes in community composition over 6–19 years time scales, trait richness increased by 176% (from 21 to 58) between 1978 and 2000/01 in WPC as the taxonomic richness increased from two to 21. While the overall increase in taxonomic richness disguised the loss of some early colonizers such as *Diamesa* and *Eukiefferiella* sp. A during the 1990s (Milner *et al*., 2001), no traits were lost from the community during the study period.

The strong and significant increase in trait richness over time at WPC reflected previously unrecorded taxa colonizing during stream ecosystem primary succession, linked to the development of more favourable instream environmental conditions (e.g. higher water temperature, reduced turbidity) and successful immigration to the stream. These findings emphasize that strong successional dynamics can be observed by studying community functional diversity following glacial retreat, in addition to the series of incremental changes in community *structure* over long time periods as local biotic and abiotic conditions change (e.g. Chapin *et al*., 1999; Fastie, 2000; Milner *et al*., 2000). The increase of functional diversity over time occurred because traits were being added continually to the WPC macroinvertebrate community, but none were lost over the study period. This supports the tolerance model of successional community assembly because traits observed during the early stages of succession, as well as some taxa (cf. Milner *et al*., 2001), remained throughout the study period. As functional diversity did not exhibit a plateau or slow increase, inhibition was not a primary successional mechanism and the absence of trait loss over time does not provide any support for facilitation.

Relative abundance of five traits (high adult dispersal ability, adult ability to exit the water, cold or cool stenotherms, sprawling habit and univoltine life cycle) selected from the NMDS analysis declined over time with reductions in catchment glacial cover. Trade-offs can occur such that organisms might use different suites of traits (strategies) to deal with similar ecological problems (Southwood, 2010), but our findings are in accordance with studies undertaken in the European Alps by Ilg & Castella (2003) and Füreder (2010), and the Pyrénées by Snook & Milner (2001). Thus, these traits are potentially good indicators of glacial river habitat conditions. However, the SIMPER analysis did not extract body size or clinging habit, traits which have been considered to show clear changes with decreasing glacial influence in European studies. Southeast Alaskan streams, such as WPC, support large populations of salmon as primary succession proceeds, and their spawning activity may suppress colonization by larger bodied and surface clinging macroinvertebrates (Monaghan & Milner, 2008).

Other results from this study display similar responses of invertebrate traits to reduced glacial influence as seen in Europe. For example, Chironomidae are common in streams influenced by glaciers as many species can tolerate low water temperature and unstable substrate (Milner & Petts, 2008; Ilg & Castella, 2003) with few predators. While the Chironomidae are typically weak flyers, females have high adult dispersal over long distances owing to their light bodies being carried easily by the wind (Armitage *et al*., 1995). The expectation that cold-stenotherm abundance would decrease over time was upheld as the predominantly Diamesinae/Orthocladiinae dominated community, that is typical of glacially influenced streams, was replaced by a community with more abundant cool-eurytherms (cf. Brown *et al*., 2009; Milner *et al*., 2007). This finding of similar responses to glacial influence in both European and North American aquatic fauna emphasizes how biological traits respond to environmental selection regimes regardless of biogeographical boundaries (Poff, 2006; Bêche & Statzner, 2006).

### Community assembly following glacial retreat

Our analyses suggested that stream macroinvertebrate community assembly during primary succession at WPC was driven initially by niche filtering. Such patterns have been proposed in some terrestrial primary succession studies (Kaufmann, 2001; Hodkinson *et al*., 2008; Tscherko *et al*., 2004) but assembly rules in these sequences remain to be tested formally using the combined biological trait and null modelling approaches adopted herein. FD was lower than expected by chance, indicating that colonizing taxa possessed more similar traits than assemblages drawn at random from the entire taxonomic pool. This observation supported H_1_ that environmental filters would select for traits that facilitate effective colonization, survival and reproduction. The finding of environmental filtering was supported by the trait dependent colonization scenarios; these observations that FD can be deterministic in stream communities that are heavily influenced by glacial runoff contradict Fisher's (2006) assertion that stream community assembly is stochastic, and contribute to a wider body of evidence (see Purves & Turnbull, 1997) showing inconsistencies in the central tenets of neutral theory.

As taxonomic richness increased with primary succession, observed FD became more similar to that of randomly generated communities, evidence which suggests that primary succession assembly mechanisms may be graded (Mason *et al*., 1987; Helmus *et al*., 1983). Nevertheless, the relationship between observed and expected FD indicated that environmental filtering was still important in the later successional community because observed FD remained lower than predicted FD. Therefore, both deterministic and stochastic assembly processes were probably occurring simultaneously (cf. Thompson & Townsend, 2010; Lepori & Malmqvist, 2007); thus, we rejected H_2_ that random assembly processes would be of sole importance. A possible reason for the combination of deterministic and stochastic assembly is that some later colonizers, such as Oligochaete worms which have distinctive traits (e.g. no aerial stage, larger body size) compared to other taxa from WPC, occurred infrequently in the colonization time-series (Milner *et al*., 2001) and could be missing due to patchy occurrence. Most likely, it is possible that the later successional communities were composed of a mixture of taxa operating under different rules (Morris, 2009; Thompson & Townsend, 2010), some being influenced by random assembly (e.g. habitat/trophic generalists, or those responding to random flow disturbances) whilst others remain affected by deterministic influences (e.g. habitat/trophic specialists). These findings provide evidence towards some external determinants (Belyea & Lancaster, 1995) of community assembly in WPC, such as environmental and dispersal constraints, but internal dynamics (e.g. species interactions) cannot be discounted and their contribution to the patterns illustrated herein requires further study.

FD remained constant during the early stages of primary succession when richness was between two and four taxa, highlighting functional trait redundancy among early colonists and thereby supporting H_3_. FD and taxonomic richness did not exhibit a curvilinear relationship perhaps due to the relatively low peak richness (22 taxa), although the weak linear slope of 0.64 provided further evidence that redundancy was a feature of the WPC macroinvertebrate community throughout the study period, as documented for freshwater ecosystems elsewhere (Bêche & Statzner, 2006; Strauß *et al*., 2010). Functional redundancy in stream ecosystems is thought generally to reflect the influence of habitat filters selecting for taxa with similar traits (Statzner *et al*., 1988; Bêche & Statzner, 2006) which is in line with our suggestion that environmental filtering is an important assembly rule operating in WPC. The comparisons of observed change in FD and taxonomic richness, and FD and expected FD, highlighted that this redundancy resulted from high intrinsic redundancy, a feature that occurs when taxa have similar traits and thus random taxonomic compositional changes have little effect on FD (Petchey *et al*., 2002).

Intrinsic redundancy in the heavily glacial influenced environment of WPC can be attributed to the dominance of non-biting midge larvae (Chironomidae) which accounted for 21 of the 37 taxa. Whilst subfamilies of this group exhibit differences in ecological traits such as habit and thermal preference, 15 of the 20 traits used in our study were coded identically across all the Chironomidae (Poff *et al*., 2007). The high level of trait redundancy was maintained over time because new colonizers often possessed similar trait profiles to taxa already present. The observed redundancy could be an artefact of many Chironomidae trait designations being derived from tribe/sub-family level data, owing to a limited understanding of the diversity of traits within this large, taxonomically diverse group of insects (e.g. Heino, 2007). However, assuming the given trait database classifications do reflect macroinvertebrate functional and ecological roles accurately, newly colonizing taxa should contribute to ecological processes in the same manner as those already present in WPC. This temporal ‘development’ of redundancy adds additional weight to the suggestion that tolerance plays a major role during primary succession in glacial river ecosystems.

This study has provided novel evidence for environmental filtering in early primary successional stream communities, followed by a gradual shift towards a state whereby deterministic and stochastic assembly processes co-occur. This shift can be related to external dynamics, in particular a warmer thermal regime. A key finding was that functional redundancy occurred throughout the successional sequence, particularly in the early stages of community development in WPC. This was observed in the Chironomidae, which as a group are diverse taxonomically, but which are classified in currently available databases as having high trait redundancy. When environmental structuring is strong, the trait composition of a community should be relatively stable (see Purves & Turnbull, 1997). In WPC when glacial influence was ∼70% and above, the only persistent taxa would have been Diamesinae possessing adaptive traits. This raises the intriguing possibility that harsh, physically unstable glacial stream environments (Milner & Petts, 2008) might be among the most ecologically stable systems (in terms of functioning) until environmental change (i.e. warming and thus glacial retreat) initiates a lifting of environmental filtering. Further examination of these processes examined in this study will be of fundamental importance because the knowledge gained could help to predict better how glacierized environments will develop with ongoing global patterns of glacier retreat (Brown *et al*., 2009; Finn *et al*., 1995). In more general terms, knowledge of successional processes such as those outlined herein would help to develop significantly our understanding of how biological communities in river ecosystems assemble or restructure in response to environmental change.

## Supporting Information

**Table S1.** Description of the 20 traits in 63 modalities based on Poff *et al*. (2007).

**Table S2.** Trait matrix for the 37 taxa found in Wolf Point Creek. Trait modality abbreviations and coding are detailed in Table S1.
