# Supplementary material for: Rapid loss of glacial ice reveals stream community assembly processes
Source: Glob Chang Biol. 2012 Mar 26;18(7):2195–204. doi: 10.1111/j.1365-2486.2012.02675.x (PMC3664027; doi:10.1111/j.1365-2486.2012.02675.x)
Supplement: Supplementary file 1 [file gcb0018-2195-SD1.docx]

**Table S1**. Description of the 20 traits in 63 modalities based on Poff et al. (2006)

| **Trait** | **Trait modality** | **Code** |
| --- | --- | --- |
| **Life history** |  |  |
| *Voltinism* | Semivoltine (<1 generation/y) | Volt1 |
|  | Univoltine (1 generation/y) | Volt2 |
|  | Bi- or multivoltine (>1 generation/y) | Volt3 |
| *Development* | Fast seasonal | Devl1 |
|  | Slow seasonal | Devl2 |
|  | Non seasonal | Devl3 |
| *Synchronization of emergence* | No emergence | Sync0 |
|  | Poorly synchronized (wk) | Sync1 |
|  | Well synchronized (d) | Sync2 |
| *Adult life span* | No adult life stage | Life0 |
|  | Very short (<1 wk) | Life1 |
|  | Short (<1 mo) | Life2 |
|  | Long (>1mo) | Life3 |
| *Adult ability to exit* | Absent (not including emergence) | Exit1 |
|  | Present | Exit2 |
| *Ability to survive desiccation* | Absent | Desi1 |
|  | Present | Desi2 |
| **Mobility** |  |  |
| *Female dispersal* | Non-insect (no adult dispersal) | Disp0 |
|  | Low (<1 km flight before laying eggs) | Disp1 |
|  | High (>1 km flight before laying eggs) | Disp2 |
| *Adult flying strength* | No aerial stage | Flgt0 |
|  | Weak (e.g. cannot fly into light breeze) | Flgt1 |
|  | Strong | Flgt2 |
| *Occurrence in drift* | Rare (catastrophic only) | Drft1 |
|  | Common | Drft2 |
|  | Abundant | Drft3 |
| *Maximum crawling rate* | Very low (<10 cm/h) | Crwl1 |
|  | Low (<100 cm/h) | Crwl2 |
|  | High (>100 cm/h) | Crwl3 |
| *Swimming ability* | None | Swim1 |
|  | Weak | Swim2 |
|  | Strong | Swim3 |
| **Morphology** |  |  |
| *Attachment* | None (free-ranging) | Atch1 |
|  | Some (sessile/sedentary) | Atch2 |
|  | Both | Atch3 |
| *Armoring* | None (soft-bodied forms) | Armr1 |
|  | Poor (heavily sclerotized) | Armr2 |
|  | Good (e.g. some cased caddisflies) | Armr3 |
| *Shape* | Streamlined (flat fusiform) | Shpe1 |
|  | Not streamlined (cylindrical, round or bluff) | Shpe2 |
| *Respiration* | Tegument | Resp1 |
|  | Gills | Resp2 |
|  | Plastron/spiracle (aerial) | Resp3 |
| *Size at maturity* | Small (<9mm) | Size1 |
|  | Medium (9–16 mm) | Size2 |
|  | Large (>16 mm) | Size3 |
| **Ecology** |  |  |
| *Rheophily* | Depositional only | Rheo1 |
|  | Depositional and erosional | Rheo2 |
|  | Erosional | Rheo3 |
| *Thermal preference* | Cold stenothermal or cool eurythermal | Ther1 |
|  | Cool/warm eurythermal | Ther2 |
|  | Warm eurythermal | Ther3 |
| *Habit* | Burrow | Habi1 |
|  | Climb | Habi2 |
|  | Sprawl | Habi3 |
|  | Cling | Habi4 |
|  | Swim | Habi5 |
|  | Skate | Habi6 |
| *Trophic habit* | Collector-gatherer | Trop1 |
|  | Collector-filterer | Trop2 |
|  | Herbivore (scraper, piercer and shredder) | Trop3 |
|  | Predator (piercer and engulfer) | Trop4 |
|  | Shredder (detritivore) | Trop5 |
