# Supplementary material for: Rapid loss of glacial ice reveals stream community assembly processes
Source: Glob Chang Biol. 2012 Mar 26;18(7):2195–204. doi: 10.1111/j.1365-2486.2012.02675.x (PMC3664027; doi:10.1111/j.1365-2486.2012.02675.x)
Supplement: Supplementary file 2 [file gcb0018-2195-SD2.docx]

**Table S2.** Trait matrix for the 37 taxa found in Wolf Point Creek. Trait modality abbreviations and coding are detailed in Table S1.

|  | **Life history** | | | | | | **Mobility** | | | | | **Morphology** | | | | | **Ecology** | | |  |
| --- | --- | --- | --- | --- | --- | --- | --- | --- | --- | --- | --- | --- | --- | --- | --- | --- | --- | --- | --- | --- |
| **Taxon [Order/Family]^1^** | Volt | Devl | Sync | Life | Exit | Desi | Disp | Flgt | Drft | Crwl | Swim | Atch | Armr | Shpe | Resp | Size | Rheo | Ther | Habi | Trop |
| *Diamesa* | 2 | 1 | 2 | 1 | 2 | 1 | 2 | 1 | 3 | 1 | 1 | 1 | 1 | 2 | 2 | 1 | 2 | 1 | 3 | 1 |
| *Brillia* | 2 | 1 | 2 | 1 | 2 | 1 | 2 | 1 | 3 | 1 | 1 | 1 | 1 | 2 | 2 | 1 | 2 | 1 | 1 | 3 |
| *Pseudodiamesa* | 2 | 1 | 2 | 1 | 2 | 1 | 2 | 1 | 3 | 1 | 1 | 1 | 1 | 2 | 2 | 1 | 2 | 1 | 3 | 1 |
| *Chaetocladius* | 2 | 1 | 2 | 1 | 2 | 1 | 2 | 1 | 3 | 1 | 1 | 1 | 1 | 2 | 2 | 1 | 2 | 1 | 1 | 1 |
| *Cricotopustremulus* | 2 | 1 | 2 | 1 | 2 | 1 | 2 | 1 | 3 | 1 | 1 | 1 | 1 | 2 | 2 | 1 | 2 | 1 | 1 | 3 |
| *Eukieferiellabrehmi* | 2 | 1 | 2 | 1 | 2 | 1 | 2 | 1 | 3 | 1 | 1 | 1 | 1 | 2 | 2 | 1 | 2 | 1 | 1 | 1 |
| *Eukieferiellaclaripennis* | 2 | 1 | 2 | 1 | 2 | 1 | 2 | 1 | 3 | 1 | 1 | 1 | 1 | 2 | 2 | 1 | 2 | 1 | 1 | 1 |
| *Eukieferiellacyanea* | 2 | 1 | 2 | 1 | 2 | 1 | 2 | 1 | 3 | 1 | 1 | 1 | 1 | 2 | 2 | 1 | 2 | 1 | 1 | 1 |
| *Eukieferielladevonica* | 2 | 1 | 2 | 1 | 2 | 1 | 2 | 1 | 3 | 1 | 1 | 1 | 1 | 2 | 2 | 1 | 2 | 1 | 1 | 1 |
| *Eukieferiellagracei* | 2 | 1 | 2 | 1 | 2 | 1 | 2 | 1 | 3 | 1 | 1 | 1 | 1 | 2 | 2 | 1 | 2 | 1 | 1 | 1 |
| *Eukieferiellarectangularis* | 2 | 1 | 2 | 1 | 2 | 1 | 2 | 1 | 3 | 1 | 1 | 1 | 1 | 2 | 2 | 1 | 2 | 1 | 1 | 1 |
| *Tokunagaia* | 2 | 1 | 2 | 1 | 2 | 1 | 2 | 1 | 3 | 1 | 1 | 1 | 1 | 2 | 2 | 1 | 2 | 1 | 1 | 1 |
| *Eukieferiella*sp A | 2 | 1 | 2 | 1 | 2 | 1 | 2 | 1 | 3 | 1 | 1 | 1 | 1 | 2 | 2 | 1 | 2 | 1 | 1 | 1 |
| *Eukieferiellatveta* | 2 | 1 | 2 | 1 | 2 | 1 | 2 | 1 | 3 | 1 | 1 | 1 | 1 | 2 | 2 | 1 | 2 | 1 | 1 | 1 |
| *O. Orthocladius* G | 2 | 1 | 2 | 1 | 2 | 1 | 2 | 1 | 3 | 1 | 1 | 1 | 1 | 2 | 2 | 1 | 2 | 1 | 1 | 1 |
| *Orthocladiusmallochi* | 2 | 1 | 2 | 1 | 2 | 1 | 2 | 1 | 3 | 1 | 1 | 1 | 1 | 2 | 2 | 1 | 2 | 1 | 1 | 1 |
| *Orthocladiusmanitobensis* | 2 | 1 | 2 | 1 | 2 | 1 | 2 | 1 | 3 | 1 | 1 | 1 | 1 | 2 | 2 | 1 | 2 | 1 | 1 | 1 |
| *Pagastiapartica* | 2 | 1 | 2 | 1 | 2 | 1 | 2 | 1 | 3 | 1 | 1 | 1 | 1 | 2 | 2 | 1 | 2 | 1 | 3 | 3 |
| *Paratrichocladius* | 2 | 1 | 2 | 1 | 2 | 1 | 2 | 1 | 3 | 1 | 1 | 1 | 1 | 2 | 2 | 1 | 2 | 1 | 1 | 1 |
| *Potthastia* | 2 | 1 | 2 | 1 | 2 | 1 | 2 | 1 | 3 | 1 | 1 | 1 | 1 | 2 | 2 | 1 | 2 | 1 | 3 | 1 |
| *Micropsectra/Tanytarsus* | 2 | 1 | 2 | 1 | 2 | 1 | 2 | 1 | 3 | 1 | 1 | 1 | 1 | 2 | 2 | 1 | 2 | 2 | 3 | 4 |
| *Suwalliaforcipata*[Plecoptera:Chloroperlidae] | 2 | 2 | 2 | 2 | 1 | 1 | 1 | 1 | 2 | 2 | 2 | 1 | 1 | 2 | 1 | 2 | 2 | 2 | 4 | 4 |
| *Baetis*[Ephemeroptera:Baetidae] | 3 | 1 | 1 | 1 | 1 | 1 | 1 | 1 | 3 | 1 | 3 | 1 | 1 | 1 | 2 | 1 | 2 | 2 | 5 | 1 |
| Simuliidae [Diptera:Simuliidae] | 3 | 1 | 2 | 1 | 1 | 1 | 1 | 2 | 2 | 2 | 1 | 2 | 1 | 2 | 1 | 1 | 3 | 2 | 4 | 2 |
| *Limnophila* [Diptera:Tipulidae] | 2 | 2 | 1 | 2 | 1 | 2 | 1 | 1 | 1 | 2 | 1 | 1 | 1 | 2 | 2 | 2 | 2 | 2 | 3 | 5 |
| Oligochaeta | 3 | 3 | 0 | 0 | 1 | 1 | 0 | 0 | 2 | 3 | 1 | 1 | 1 | 2 | 1 | 3 | 2 | 1 | 1 | 1 |
| Ceratopogoniidae [Diptera:Ceratopogonidae] | 2 | 1 | 1 | 1 | 1 | 1 | 1 | 1 | 1 | 1 | 1 | 1 | 2 | 2 | 1 | 1 | 2 | 2 | 3 | 4 |
| *Onocosmoecus* [Trichoptera:Limnephilidae] | 2 | 2 | 2 | 2 | 1 | 1 | 1 | 1 | 1 | 2 | 1 | 2 | 3 | 2 | 2 | 3 | 1 | 1 | 3 | 5 |
| *Ecclisomyia*[Trichoptera:Limnephilidae] | 1 | 2 | 2 | 2 | 1 | 1 | 1 | 1 | 1 | 2 | 1 | 2 | 3 | 2 | 2 | 2 | 3 | 1 | 4 | 3 |
| Gammaridae [Amphipoda:Gammaridae] | 3 | 1 | 0 | 0 | 1 | 2 | 0 | 0 | 3 | 1 | 3 | 1 | 2 | 2 | 2 | 3 | 3 | 2 | 5 | 5 |
| Dytiscid larvae [Coleoptera:Dytiscidae] | 1 | 2 | 1 | 3 | 2 | 1 | 2 | 2 | 1 | 3 | 3 | 1 | 3 | 1 | 3 | 2 | 2 | 2 | 5 | 4 |
| Empididae [Diptera:Empididae] | 2 | 2 | 2 | 1 | 2 | 1 | 1 | 1 | 1 | 2 | 1 | 1 | 1 | 2 | 1 | 2 | 2 | 2 | 3 | 4 |
| *Hesperophylax*[Trichoptera:Limnephilidae] | 2 | 2 | 2 | 2 | 1 | 1 | 1 | 1 | 1 | 2 | 1 | 2 | 3 | 2 | 2 | 3 | 2 | 2 | 3 | 5 |
| Perlidae [Plecoptera:Perlidae] | 1 | 2 | 1 | 2 | 1 | 1 | 2 | 2 | 1 | 3 | 2 | 1 | 2 | 1 | 2 | 3 | 3 | 1 | 4 | 4 |
| *Taeniopteryx*[Plecoptera: Taeniopterygidae] | 2 | 1 | 2 | 1 | 1 | 2 | 1 | 1 | 1 | 1 | 2 | 1 | 1 | 2 | 2 | 2 | 2 | 2 | 3 | 5 |
| *Kathroperla* [Plecoptera:Chloroperlidae] | 2 | 2 | 2 | 2 | 1 | 1 | 1 | 1 | 1 | 2 | 2 | 1 | 2 | 2 | 1 | 3 | 3 | 1 | 4 | 1 |
| *Brachycentrus* [Trichoptera:Brachycentridae] | 2 | 2 | 2 | 2 | 1 | 1 | 1 | 1 | 1 | 1 | 1 | 3 | 3 | 2 | 2 | 2 | 3 | 2 | 4 | 2 |

^1^Order:FamilyisDiptera:Chironomidae except where specified, and for Oligochaeta which were identified only to Order
